# Expert voices and equal partnerships: establishing Controlled Human Infection Models (CHIMs) in Vietnam

**DOI:** 10.12688/wellcomeopenres.15337.1

**Published:** 2019-09-25

**Authors:** Evelyne Kestelyn, Chi Le Phuong, Jennifer Ilo Van Nuil, Hoai Tam Dong Thi, Nguyet Minh Nguyen, Trung Dinh The, Mary Chambers, Cameron P. Simmons, Toan Nguyen Trong, Dung Nguyen The, Le Truc Phuong, Dung Do Van, Dung Duc Anh, Vinh Chau Nguyen Van, Stephen Baker, Bridget Wills, OUCRU Controlled Human Infection Models workshop and focus group participants

**Affiliations:** 1 Oxford University Clinical Research Unit, University of Oxford, Ho Chi Minh City, Vietnam; 2 Center for Tropical Medicine and Global Health, University of Oxford, Oxford, UK; 3 Institute for Vector-Borne Disease, Monash University, Clayton, VIC 3800, Australia; 4 Pasteur Institute, Ho Chi MInh City, Vietnam; 5 Department of Infectious Diseases, School of Medicine, Vietnam National University, Ho Chi Minh City, Vietnam; 6 Nguyen Tri Phuong Hospital, Ho Chi Minh City, Vietnam; 7 University of Medicine and Pharmacy, Ho Chi Minh City, Vietnam; 8 Hospital for Tropical Diseases, Ho Chi Minh City, Vietnam

**Keywords:** Human Challenge Studies, Controlled human infection model, CHIM, vaccine, workshop report

## Abstract

The number of controlled human infection models (CHIMs) conducted worldwide has increased considerably in recent years, although few have been conducted in low and middle-income countries (LMICs), where infectious diseases have the greatest burden. Recently Oxford University Clinical Research Unit (OUCRU) in Ho Chi Minh City (HCMC) started developing CHIM research proposals motivated by the need to develop a clearer and more grounded understanding of the issues surrounding the conduct of CHIMs in LMICs. To explore initial perceptions and barriers to conducting CHIMs in Vietnam, OUCRU researchers conducted a set of key stakeholder interviews early in 2018 and held a CHIM workshop in HCMC in March 2018. This paper summarizes the discussions from the workshop and outlines a way forward for conducting CHIMs in Vietnam.

## Introduction

The development of new vaccines for infectious diseases is an area of unmet need in global health as highlighted by the emergence of various highly pathogenic influenza viruses over the last 20 years and by the more recent outbreaks of Zika and Ebola ^1– 3^. These global threats have spurred big donors, such as Wellcome, the Medical Research Council (MRC), the Bill & Melinda Gates Foundation, and Horizon 2020 to invest in controlled human infection models (CHIMs) in which healthy volunteers are intentionally infected with a pathogen. Such studies have a long history and are increasingly being exploited to fast track the development of new therapeutics and vaccines for infectious diseases and to generate new insights into disease pathogenesis ^4– 7^. Despite a contentious past, CHIMs can be a safe and highly cost-effective mechanism to study infectious diseases, particularly those for which no animal model exists ^1, 3, 5^. The number of CHIMs conducted worldwide has increased considerably in recent years, although few have been conducted in low and middle-income countries (LMICs), where infectious diseases have the greatest burden ^3, 8, 9^.

A common disease that plagues many LMICs is dengue, an arboviral infection which exerts a major economic and social burden in tropical and sub-tropical regions ^10, 11^. Dengue virus infection is particularly prevalent across Southeast Asia and is hyperendemic in Vietnam with periodic large outbreaks superimposed on sustained annual transmission ^12^. No specific therapeutic agents are available for dengue, vector control strategies have not had a significant impact on transmission, and the licensure of an effective vaccine has been hampered by complex disease pathogenesis and the need to elicit protection against all four dengue serotypes simultaneously ^11, 13– 15^. A process known as antibody-dependent enhancement (ADE), by which low levels of antibodies elicited in response to an initial exposure to one serotype appear to increase the risk of developing severe disease when an individual is subsequently infected by another serotype, is a particular cause for concern ^16– 18^. Conducting dengue CHIMs alongside other clinical dengue research could significantly inform the down-selection of vaccine candidates and support the targeted development of potential antiviral agents for therapeutic or prophylactic indications ^13, 19^; such studies are now being performed in the United States ^19, 20^. Although the scientific and public health importance of involving populations from disease endemic settings in vaccine-related research is well known and is particularly important for dengue ^3, 7, 9^, to date, no dengue CHIM studies have been conducted in dengue-endemic settings ^6, 19^.

Recently Oxford University Clinical Research Unit (OUCRU) in Ho Chi Minh City (HCMC) started developing CHIM research proposals motivated by the need to develop a clearer and more grounded understanding of the issues surrounding the conduct of CHIMs in LMICs ^6, 7, 9, 19, 21^. Due to the considerable health and economic burden of dengue in Vietnam, the disease has been a major focus of research at OUCRU, so we decided to focus on dengue CHIM studies in particular. However, the controversial nature of conducting a human challenge with an infectious agent for which there is no therapeutic currently available and which may be associated with future, potentially serious, consequences, makes this a challenging prospect. Enteric diseases are also a key research focus at OUCRU, and since endemic setting *Shigella* CHIM studies are currently under consideration by the global health community, we used *Shigella* CHIMs as another example, aiming to broaden the discussion to include important issues specific to enteric pathogens.

To explore initial perceptions and barriers to conducting CHIMs in Vietnam, OUCRU researchers conducted a set of key stakeholder interviews early in 2018 and held a CHIM workshop in HCMC in March 2018. This workshop gathered CHIM researchers from across Southeast Asia together with key OUCRU collaborators, aiming to explore and discuss the scientific, ethical, and practical issues related to conducting human challenge studies in Vietnam. This paper summarizes the discussions from the workshop and outlines a way forward for conducting CHIMs in Vietnam.

## Methods

### Selection of participants and workshop format

**Figure 1.**
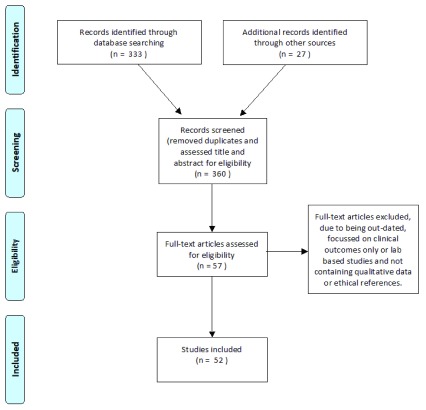
Modified PRISMA flow diagram.

At the 6 ^th^ annual Global Health Bioethics Network (GHBN) meeting held in Durban, South Africa, in September 2017 we presented and discussed pertinent ethical issues regarding the implementation of CHIMs in LMICs ^22^. Meeting participants included ethics and engagement staff from the Wellcome Trust Africa and Asia Programmes in Kenya, Malawi, South Africa, Thailand, Laos, and Vietnam (and partner sites in Nepal and Indonesia), members of the Ethox Centre team in Oxford, and representatives from the Wellcome Trust Brighton and Sussex Centre for Global Health Research. We grouped participants to discuss predefined questions around public engagement, reimbursement and consent and encouraged them to explore and reflect on main ethical issues in CHIMs ( Box 1). We collated themes from that meeting with themes identified through a literature review conducted between October and December 2017. This initial review was mostly based on a PubMed search using a modified PRISMA diagram ( Figure 1). Search terms used in title/abstract (added filter for “human studies”) were:

Box 1. **Predefined questions to guide group discussion at the Global Health.**

Bioethics Network meeting in South Africa, in September 2017. CHIM: controlled human infection models

**Pre-defined questions to guide group discussions.**

We would like you to discuss (in a group) one of the following issues and tie that in with “What would be interesting/useful ethics research projects to be done alongside CHIM studies?”

- What do they think are likely to be the main consent related challenges in CHIM studies and how might they be addressed? ^1^
- Should participants in CHIM studies be compensated? If so, how should the amount be decided? ^1^
- What would be innovative ways of doing community engagement around the ethics of CHIM? ^1^
- What are the main ethical issues arising from CHIM studies? ^2^

“Controlled Human Infection Models”, “Human Challenge Trials”, “Dengue Vaccine Trials”, “Shigella Vaccine Trials”, “Human Challenge Models” “Human Challenge studies”, and “Ethics and Infection Models”. This yielded 333 results to which we added 27 articles identified through the reference lists of the PubMed papers as well as recommendations from colleagues and collaborators. We screened titles and abstracts for eligibility and after removing duplicates, we reviewed 57 full-text papers of which 52 were included in our review to identify relevant themes.

Together these themes formed the framework for the focus group and interview guides used in a series of focus group discussions (FGD) and interviews held in January and February 2018 with selected stakeholders. We identified stakeholders through contacting senior staff from OUCRU collaborating hospitals (the Hospital for Tropical Diseases, the Cho Ray Hospital, the Pham Ngoc Thach Hospital, Children Hospital 1 and 2), the Pasteur Institute and two of the major medical schools in the city. We asked to propose a small panel of interested staff members from their institutions for the FGDs. The director of the National Institute for Hygiene and Epidemiology in Hanoi also contributed his time for an interview. These discussions provided an initial impression of local attitudes towards CHIMs and were the initial steps in gauging the feasibility of conducting CHIMs in Vietnam.

We additionally organized a consultative workshop, entitled “Expert Meeting to Explore the Potential for Human Challenge Studies in Vietnam”, on 1-2 March 2018, which brought together key individuals from Vietnamese government and research institutions, ethics committees, universities, and major collaborating hospitals, as well as researchers currently involved in CHIM studies in SEA. We opened the workshop with general presentations describing CHIM methodologies and related ethical considerations, followed by a general Q&A session. On the first afternoon we focused the presentations on *Shigella*, leading up to group discussions led by a facilitator using pre-defined questions based on the World Café concept ^23^. On the second day, we held a series of presentations that highlighted the particular issues associated with dengue CHIMs, again followed by diverse group discussions each led by a facilitator using pre-defined questions ( Box 2). In the final session, we used dengue and *Shigella* case studies to facilitate a general debate regarding the major issues surrounding future CHIM studies in Vietnam. We obtained verbal consent from all participants for both key informant interviews and the workshop to record and publish the findings of the meetings.

Box 2. **Predefined questions used by facilitators to guide group discussions at the “Expert Meeting to Explore the Potential for Human Challenge Studies in Vietnam”. CHIM: controlled human infection models.**

Pre-defined questions used for the group discussions on dengue CHIMs.

1. Is it possible to implement a dengue CHIM in Vietnam? (Advantages/barriers)
2. What are the main ethical issues you care about if a dengue CHIM is established in Vietnam?
3. What are the benefits and risks of CHIM?
4. What should we do to engage with the Ministry of Health to implement a dengue CHIM in Vietnam?
5. What should we do to engage with the community when implementing a dengue CHIM in Vietnam?
6. Which populations should be included/excluded when implementing a dengue CHIM in Vietnam?

Pre-defined questions used for the group discussions on shigella CHIMs.

1. Is Vietnam a suitable location for a shigella challenge study?
2. How should volunteers be compensated?
3. Are there specific populations to exclude/include?
4. What are the main ethical issues regarding CHIMs in Vietnam?
5. What community engagements do we require?
6. What are the ethical considerations of infecting a healthy Vietnamese person with a pathogen?
7. What is the risk to the public of CHIMs in Vietnam?
8. How do we engage with the Ministry of Health to establish CHIM guidelines?

### Data management and analysis

**Figure 2.**
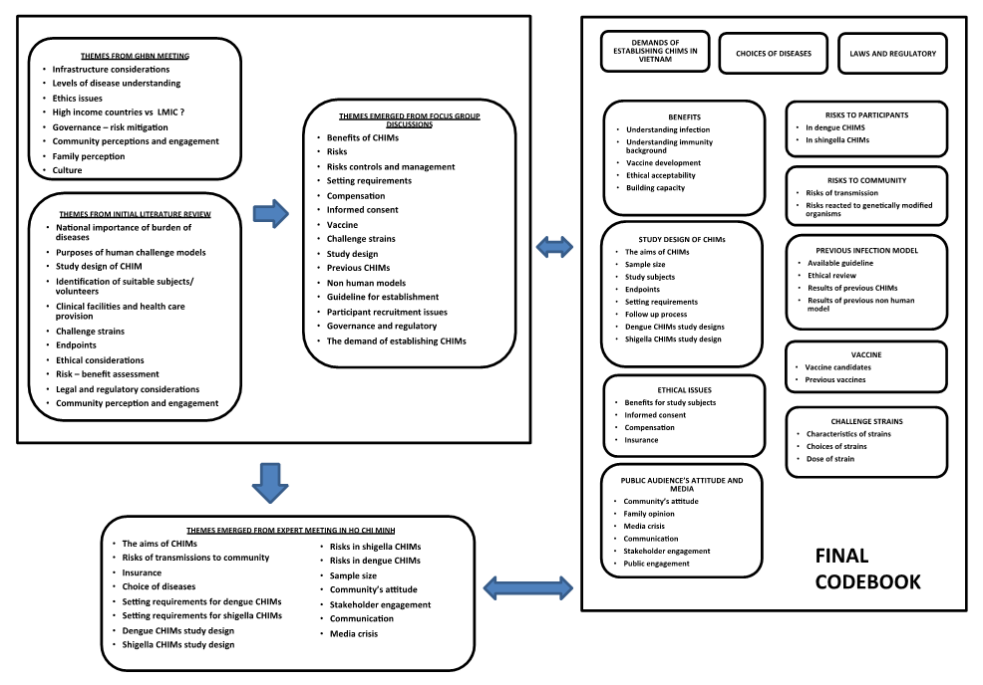
Controlled human infection models (CHIM) codebook. The processes of developing a finalized codebook are shown in the figure. We developed an initial codebook using the key themes identified from a Global Health Bioethics Network meeting and our literature review. Themes emerging from the focus group discussions and the CHIM workshop in Vietnam were incorporated. We also created open codes for excerpts not covered by the closed codes leading to the finalized CHIM codebook. LMIC: low-to-middle-income countries.

**Figure 3.**
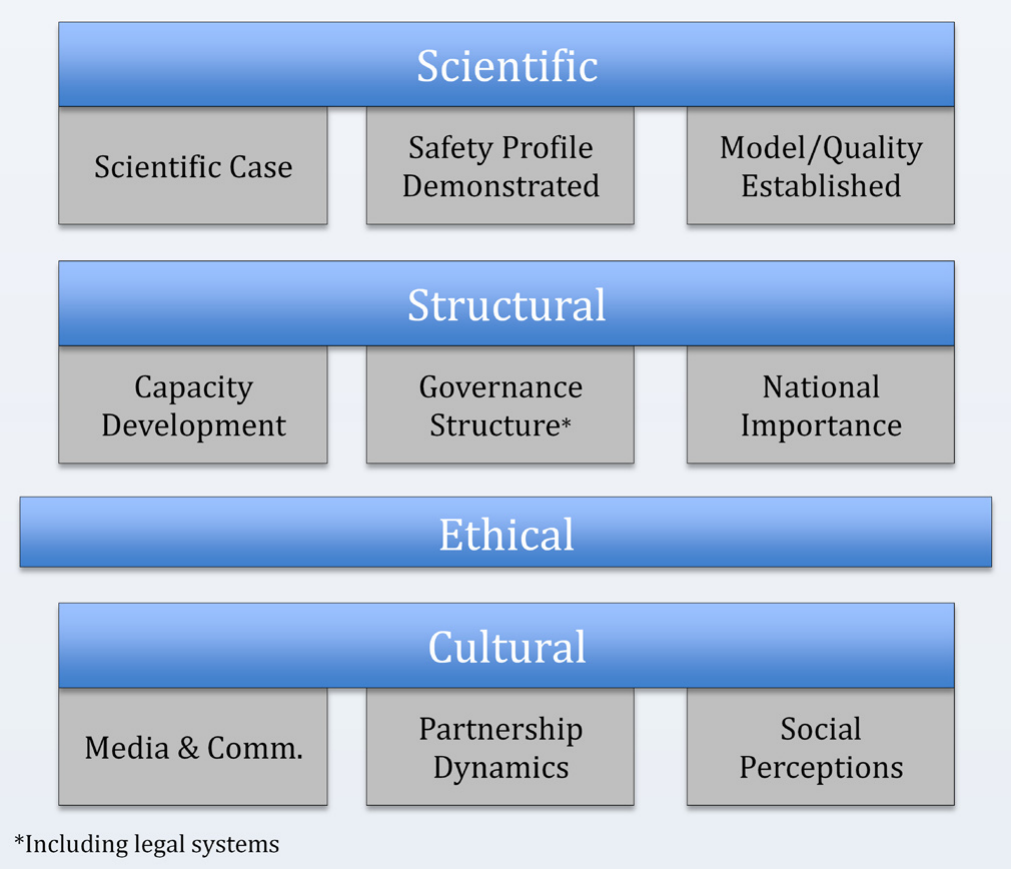
An initial framework for controlled human infection models (CHIM) in Vietnam. A framework to guide future CHIM work in Vietnam building on the Malawi framework and based on the discussions and the findings of our workshops. This framework is based on four main concepts – Scientific, Structural, Ethical and Cultural – each encompassing a number of different themes or issues to consider.

We recorded the discussions through handwritten notes for the key stakeholder interviews and using a voice recorder at the workshop. Discussion notes were typed and transcribed, digital recordings were translated where necessary and uploaded into Nvivo 12. According to OUCRU standard operating procedures, all files, including audio files will be kept for a minimum of three years. Audio and/or video files used for informed consent purposes will be kept for a minimum of 15 years. These electronic files will be stored in an appropriate secure, restricted access folder found on the OUCRU-VN server. For coding and analysis, we used a top-down version of thematic analysis, which involved multiple readings of the data while coding and recoding the transcripts using elaborative coding techniques as described by Auerbach & Silverstein ^24^. We developed an initial codebook using the key themes identified from both the GHBN meeting and our initial literature review. We also created open codes for excerpts not covered by the closed codes, related to the feasibility of conducting CHIMs in Vietnam and LMICs generally, as well as issues regarding long-term risks and controlling onward dengue transmission via mosquitoes. Throughout the process, we edited the meaning of the themes by reducing or expanding the description as required and then grouped the codes into larger thematic categories ( Figure 2). Finally, we organized the themes into theoretical constructs building on the conceptual framework developed at a CHIM workshop in Malawi ^9^. This became the foundation for the elaborated framework we developed based on the data ( Figure 3).

## Results

A series of 4 FGDs involving 25 participants, plus 5 interviews with key stakeholders, were held in January and February 2018. Subsequently, 51 participants from 4 countries (UK, Singapore, Australia, and Vietnam), with a range of different backgrounds, expertise and perspectives attended the workshop in HCMC in March 2018. The findings from these various activities are presented according to the overall themes that we identified during the analysis, with general observations presented first followed by disease specific comments later when relevant ( Table 1). Given the significance of dengue in the Vietnamese public health arena, and the perceived complexity of dengue CHIM studies, the majority of the discussions revolved around potential dengue CHIM research. However, we think that the problems identified and the general principles articulated, are relevant across the spectrum of CHIM studies involving infectious agents that might be performed in LMIC settings.

**Table 1.** Summary of major topics and subthemes. Summary of the overall themes identified illustrated with selected quotes.

Community benefits and risks	
Facilitating development of vaccines and therapeutics	*“That is an important point, especially for conditions being common in endemic areas, for which* *there are not enough sites to do Phase 3 studies for every vaccine.”* *“This type of research has potential when thinking about vaccine development.”*
Pathogenesis research	*“This model will allow us to explore why this person has severe illness, while the others have mild* *illness.”*
The choice of challenge strains and their characteristics	*“Yes, it is the difficulty we have. You have to prove that if you infect with the wild type and the* *experimental type, they have the same risks, the same virulence.”*
Building and strengthening scientific and research capacity	*“Building capacity involves developing clinical sites, laboratory sites and also regulatory and ethical* *aspects if they have wide relevance… in that way we see that we feel fascinated in investing in low* *and middle income countries.”*
Potential secondary transmission and community exposure	*“Screening people daily to be sure that they will not share the organisms when they leave. We* *cannot let people leave until we know that there is no risk for transmission to the community.”*
Participant motivations and risks	
Individual participant risk	*“What about ethical issues? Do you think that exposing people to potentially dangerous pathogens* *is acceptable? There is a risk, a small risk, but it still exists, maybe minor than in other clinical trials* *or not.”*
Immediate versus long-term risks	*“They have been challenged, then they are healthy, and they go. Maybe a year or two later, they go* *for a holiday in some isolated islands and they get secondary infection in a place where they have* *no health care. That would be a risk.”*
Reimbursement and risk compensation	*“Personally, I may join the study for altruism. Maybe, or someone else. But how we can be sure that this* *is the true altruism? I think it is the most challenging question to me.”*
Selection and consent of study participants	*“Should we allow people to take that risk if they really understand it? Who makes the decision?* *Should it be individuals to make decisions or should it be authorities?”*
Barriers and challenges	
Regulatory and legal context	*“How can these studies be conducted in Vietnam? And what we can do for that? You know that* *in Vietnam if you want to do anything related to human research, you must follow the regulations,* *the laws, the guidelines from MoH… I would like to propose OUCRU and the international experts,* *after this workshop, we can work closely together to prepare and develop the guidelines for human* *challenge studies in Vietnam.”*
Ethical framework	*“National Guidelines for Ethics in Biomedical Research were releases by MoH. In vaccine trials, this* *guideline requires vaccines to be tested in three phases. This guideline does not mention human* *challenge studies.”*
Media and public perception	*“In some ways, it may depend on what the article says, how the article represents the issues. You* *could have a journalist whose attitude is negative, or you may have a journalist who could say that* *this is a really important way to developing science to try and help Vietnamese people solve serious* *problems.”*

## Community benefits and risks

### Facilitating development of vaccines and therapeutics

All participants agreed that there were potential benefits to initiating CHIM studies in Vietnam. Notably, there would likely be an advantageous effect on vaccine development, which was one of the most compelling reasons for conducting CHIMs in the country. However, some experts expressed suspicion towards the lack of potential economic benefits that Vietnam might gain if CHIMs are established. They indicated that even if human challenge studies could be implemented successfully in Vietnam that the country does not currently have much power in negotiating vaccine prices, which are largely dependent on the policies of big pharmaceutical companies and international sponsors. In addition, there were comments that sponsors and the pharmaceutical industry could benefit financially and through accelerated product development if new interventions were developed through academic and/or public funding, but these benefits might not be passed onto the general population. However, some participants expressed the view that since vaccine candidates tested in CHIM studies are mostly those at an early stage of development, that such infection models can be used as a stop/go for vaccine candidates. This process may reduce the number of vaccines that eventually fail after entering phase III or IV clinical trials, which would be of benefit to the researchers and the population. Some discussion ensued regarding vaccines (i.e. Dengvaxia) that were brought into the market but were later identified as having important safety issues, with huge financial and human consequences as a result ^25– 27^. Overall, participants felt strongly about ensuring that the economic and scientific benefits of CHIM research are readily accessible within the country.

### Pathogenesis research

In the case of dengue, the workshop participants felt that CHIMs could contribute to major scientific advances as there are still many unknowns regarding the pathogenesis of dengue infections and the immune response to dengue viruses. A CHIM could aid in addressing some of these outstanding questions, particularly with respect to the antibody responses elicited by primary infections versus subsequent infections with a different dengue serotype. Performing such studies locally could also help to better characterize the background immunological and genetic profile of the population, thus ensuring that future treatment and prevention strategies are relevant to Vietnamese people.

### The choice of challenge strains and their characteristics

As the pathogen strains used in human challenge models are usually attenuated, some experts argued that these might not mimic natural infections well enough to elicit similar immune responses to those induced by wild-type organisms. Therefore, the efficacy of vaccine candidates tested with these viruses/strains may not reflect the situation with wild-type infections, raising concerns about broader generalization of efficacy estimates achieved from CHIMs. Furthermore, the potential risk of using genetically modified organisms was discussed briefly, with concerns raised with respect to onward transmission beyond the immediate challenge study participants, with the potential for the challenge organism to become established in the environment.

### Building and strengthening scientific and research capacity

Workshop participants felt strongly about ensuring a sustainable investment in the countries/sites where CHIMs might be conducted in terms of infrastructure, equipment, and human resources. Building capacity in research and advancing scientific progress were important and “valuable” reasons for conducting human challenge studies in Vietnam.

Participants listed several crucial requirements for implementation of CHIM including the need for highly trained and experienced healthcare staff working in well-equipped facilities with the capacity to screen potential study participants, conduct study procedures to international standards, effectively manage adverse events, and prevent secondary transmission of the challenge organisms. Potential adverse events with a *Shigella* CHIM were considered less likely to be serious and therapeutic options are available if necessary. However, many senior clinicians that were present at the meeting were confident that the local experience treating dengue was exceptional and specialists would be able to provide comprehensive supportive care for dengue and its complications, despite the fact that there is no specific intervention currently available.

### Secondary transmission and community exposure

Many attendees expressed their concerns relating to safety assurance and the need to minimize the risks of onward transmission of challenge organisms into the community. The attendees highlighted the need for highly controlled facilities to be created to prevent unintended negative consequences, such as effective waste management systems for the *Shigella* CHIM studies. In addition, they raised the possibility of onward dengue transmission if mosquitoes were to bite dengue CHIM volunteers. Mosquito screening of the challenge facility would be essential, as well as assurance that none of the volunteers had a detectable viremia before they left the study site.

## Participant motivations and risks

### Individual participant risk

Some experts indicated that despite the potential for acquisition of important scientific knowledge, human challenge studies would not bring any direct benefit to the participants. Furthermore, they considered that the level of risk may be similar to, or higher than, that incurred in vaccine trials. Participants compared CHIMs with randomized controlled trials (RCTs); trial participants receive benefits related to general healthcare and treatment for their illness (with these benefits seen as an advantage of enrolment and a justification for such research to be conducted in Vietnamese hospitals). Conversely, healthy volunteers in CHIM studies would not receive similar benefits to those enrolled in RCT. Some meeting participants also argued that ethical committees might not approve these types of studies under the criterion that personal risks for study subjects should not outweigh potential advantages to the wider community.

### Immediate versus long-term risks

According to Vietnamese clinical trial regulations and Vietnamese law, it is obligatory for the sponsor to cover payment of all medical fees for study participants separately from the national healthcare insurance scheme. There was substantial discussion around this responsibility and the potential duration of this obligation to pay all medical fees, given the considerable uncertainty surrounding the length of required follow-up for challenge studies. A follow-up duration from 3 to 5 years was discussed, which was based on previous studies and the likely period that volunteers may be willing to commit to attending study visits. However, there was concern that this period may not be adequate for dengue CHIM studies due to the potential for ADE after natural dengue infection. This issue is particularly problematic in dengue-naïve individuals at the time of challenge. Naïve individuals are predicted to have a low risk of adverse events during challenge, but may experience severe dengue due to ADE after a subsequent natural infection. Conversely, semi-immune individuals (i.e. those with evidence of prior exposure to at least one dengue serotype) could experience ADE during the challenge itself, but would be likely to have protective immunity afterwards since severe dengue is rarely encountered once an individual has experienced infection with at least 2 serotypes. The failure of Dengvaxia was discussed in this context and raised a lot of concern. The possibility of offering vaccination to dengue CHIM volunteers after the challenge was seen as one way of mitigating this risk, but there was concern that the risk window would continue indefinitely for volunteers living in dengue-endemic areas.

### Reimbursement and risk compensation

These controversial issues around reimbursement and risk compensation led to discussions about appropriate compensation for study participants and raised questions regarding how to ensure that potential participants would not join a study because of the financial incentives. Experts agreed that study participants should not bear any costs for participating and that the sponsor should cover expenses related to all study visits and commitments, including the initial screening tests even if the volunteer did not subsequently participate. Thus reimbursements should be mandatory for time and lost income, including any period of isolation during the actual challenge, as well as for all costs for travel and for the long-term follow-up commitments. The level of additional compensation for “risk” was discussed at length, but a consensus agreement was not reached.

### Selection and consent of study participants

All workshop participants agreed that the scientific gains for the wider community need to be carefully balanced against the risks for the individual study participant. Medical risks to individual participants were discussed for both dengue and *Shigella* CHIMs. These discussions centered around available clinical and epidemiological evidence for each pathogen, as well as the burden of these diseases in Vietnam.

A central part of the discussion was how to select appropriate volunteers for CHIM research. All experts agreed that study participants must be adults with the ability to fully comprehend the complexity of such research studies. Many participants felt that students could be a potential source of study participants because they generally come from a cross section of society, have a high educational background that allows for better understanding of the scientific information presented, and are typically in comparatively stable financial positions.

We had significant discussions regarding who else should be involved in the decision process to participate in a challenge study. Experts drew attention to the tensions CHIM participation could cause within a family and emphasized the need for family involvement in the decision-making process. Despite the fact that potential study volunteers would be >18 years old and have the full capacity to make decisions independently, the group emphasized that the family plays an important role and could influence the decision of volunteers to participate. Within the family, young adults are still regarded as precious children, and participants felt that family members would want to be involved in the decision to participate, especially if the research was considered to carry some risk. Therefore, there were suggestions that family members should be approached and consented, in addition to the volunteers. Healthcare staff, especially senior clinicians, were also seen as active and influential participants in the decision-making process and the expert group emphasized that researchers should engage with them before, during, and after study completion.

Specifically, it was highlighted that selection of study volunteers for dengue CHIM research must also consider differences in risk profiles between naïve and semi-immune individuals. However, there are methodological challenges in reliably measuring the dengue immune status of individuals; the most robust screening techniques are not currently available in Vietnam. Furthermore, these techniques are time consuming and technically demanding, and a collaboration with an established centre of excellence elsewhere in the region might be necessary to ensure the screening process is sufficiently robust. The performance of dengue challenge in both groups (naïve and semi-immune) would be of great interest scientifically, but the meeting participants acknowledged that the recruitment and follow-up of naïve individuals might be more difficult, given that they would likely come from rural locations rather than cities. Urban environments in the tropics facilitate the transmission of dengue, and almost all HCMC residents have some immunity by early adult life. However, recruiting naïve participants from rural areas comes with challenges related to appropriate long-term healthcare access. Meeting participants expressed concerns that study subjects may experience ADE when exposed to dengue at some future date, with considerably greater risk for those living in rural areas where the quality of health services and experience of healthcare providers are generally lower than in cities.

## Barriers and challenges

### Regulatory and legal context

All experts perceived the lack of a regulatory and legal background as one of the main barriers to establishing and conducting CHIMs in Vietnam. This was highlighted several times during the meeting as, according to Vietnamese law, the actions of intentionally spreading dangerous epidemics to humans are prohibited. Therefore, human challenge studies, by their nature i.e. infecting humans intentionally with pathogens, would seem to contradict these laws. However, Article 8 of The Law on Prevention and Control of Infectious Diseases and Article 240 of the Criminal Code of Vietnam do not clearly define which organisms can or cannot be used within the framework of clinical trials and there is no information as to what situations may be exempt from these laws ^28^. These laws led to a lot of confusion about how to determine which diseases might be considered for human challenge studies and which should not. Differences in opinion were also noted with regard to the meaning of the term “intentionally spreading’ and whether CHIM studies could be considered as spreading infectious diseases. There was also an important discussion regarding which review procedures should be applied to evaluate the products/organisms used in CHIMs. Some experts thought that vaccine candidates and challenge strains for CHIM studies should be considered as new drugs and evaluated following FDA regulations and GMP guidelines. However, other experts indicated that both vaccine candidates and challenge strains contain live attenuated organisms, which should be assessed under the regulations for vaccine trials rather than those for new drugs. All experts agreed that human challenge studies should not replace other clinical trials. Additionally, the experts felt strongly that the aims of CHIMs should be explicit, and that it would be essential to differentiate research intended to better understand disease pathogenesis from research designed to assess vaccine efficacy. Experts also stressed the importance of properly defining the phase of vaccine trials for CHIM research.

### Ethical framework

To date, the Vietnamese national guidelines for ethics in biomedical research do not include specific instructions for reviewing CHIM studies. Additional guidance focused on developing the framework and explicit criteria for ethical review in human challenge studies would be essential. Experts stated that clear guidelines for CHIM study design and endpoints were needed to minimize and control the risks, as well as to provide indicators and criteria for Ethics Committees (ECs) to review and make informed decisions about which human challenge studies should be approved. Participants indicated that even though an ethical framework similar to those reviewed in the workshop was useful, a lot of responsibility would rest on the shoulders of the local EC members and that decisions would need to be carefully considered for each individual study ^4, 5, 29^.

### Media and public perception

The role of the media and the importance of considering public perceptions of CHIM research were highlighted as topics that would need to be managed early on in the development of any CHIM. Experts raised this point on several occasions during the meeting, highlighting the importance of monitoring the public’s perception of current and future CHIM research and emphasizing the need for clear and transparent communication at every stage, alongside active and ongoing public engagement.

## CHIM framework

Building on the Malawi framework and based on the discussions and the findings of our workshops, we developed a framework to guide the development of CHIM studies in Vietnam ( Figure 1) ^9^. This framework is based on four main concepts (Scientific, Structural, Ethical, and Cultural), each encompassing a number of different themes or considerations. The scientific considerations in our framework are comparable to the Malawi themes and relate to the need for a strong scientific justification without an alternative approach, combined with the use of already established models of quality supported by robust data and with good safety profiles. Interlinked with this need to address specific scientific questions, our framework highlights the importance of incorporating structural concepts i.e. ensuring the issues focused on are in line with national research priorities, and that the research will contribute to sustainable capacity building and infrastructure development, both in terms of clinical/laboratory facilities and in relation to governance. Governance structures in these discussions were seen from a broad perspective and included regulatory aspects as well.

## Summary

There was a consensus that CHIM studies should be reviewed initially using current guidelines for medical research involving human subjects, but this was seen as a starting point and several experts described the need for additional regulatory and ethical guidelines specific to CHIM studies to be developed. The tension between scientific progress and individual benefits/risks specific to CHIM studies needs to be addressed and is especially relevant for studies involving pathogens with variable and sometimes delayed medical consequences, such as those described above for dengue and/or observed during the recent ZIKA outbreaks ^2, 7, 30, 31^. Other ethical challenges include ensuring fair recruitment and inclusion of volunteers, achieving an acceptable level of informed consent, and deciding on appropriate compensation.

Partnership dynamics are fuelled by historical and cultural components as well as social perceptions. It is apparent that more attention needs to be paid to reducing partnership disparities in many LMICs, including Vietnam. Suspicion and mistrust were voiced several times during the workshop suggesting a gap in trust in existing relationships. Trust as a key feature of how different stakeholders relate to each other in the sphere of medicine and public health is crucial to the success of achieving important goals ^32^. Building trust is essential when developing novel research ideas, but is a complex operation, contingent on many factors ^33^. It will take the commitment of multiple partners within and outside Vietnam, working together to establish a more equal scientific playing field, to counter real and perceived exploitation. We see an important role for public engagement, in which stakeholders and collaborators actively engage with each other, facilitating open discussion of delicate issues such as how best to ensure equitable access to the benefits of CHIM research in the future ^34^. Building on the roadmap proposed by the Malawi workshop we think that it is imperative to appreciate the context and history of research in LMICs and we advocate for a strong social science component in parallel to any clinical/basic science oriented CHIM studies ^35^.

We also highlight the important role of the media in shaping the discourse around CHIM research in the public domain and emphasize that early implementation of an effective communication strategy will be crucial to the success of any proposed CHIM research in Vietnam. Press and media engagement (including social media), should focus on identifying and mitigating real and perceived risks related to challenge studies, but should also monitor the influence of the increasingly powerful global anti-vaccination lobby on public opinion in the country and across the region.

Based on the feedback from this initial workshop we are adopting a stepwise approach to identify and tackle barriers and challenges to implementing CHIM research in Vietnam. In line with previous workshops held in LMICs the importance of local capacity building and infrastructure development to ensure levels of local expertise and experience was emphasized at the meeting. However, the participants were generally positive that by working closely with partners already experienced in the CHIM field, that safe and scientifically sound models could be delivered ^9, 13^. The need for comprehensive guidelines and guidance was highlighted throughout the meetings. We plan to develop a regulatory framework relevant to the Vietnamese setting and will work with our key partners to explore and where possible adapt current legislation.

## Conclusions

The consensus workshop discussions helped to identify common concerns and potential challenges to conducting CHIM research in Vietnam. Dengue remains a major burden for public health services in the country and was therefore considered to be an important pathogen for potential CHIM research; all participants felt that developing effective interventions to prevent and manage dengue will remain a high priority within the national research agenda. The development of a framework that is suitable for dengue CHIMs would likely also permit performance of challenge studies using other pathogens that are of interest to the Vietnamese public-health community. Every pathogen presents a particular combination of ethical and practical issues for consideration but, given the unusual complexity of dengue immunology and risk profiling, it is likely that a framework that in principle allows a dengue CHIM to proceed would also be suitable for other pathogens. It is essential to include the voices of local scientists and clinicians in developing these projects. However, we recognize the need to involve the wider health community and the public in making decisions that could potentially have a broader impact on society than more conventional medical research. The next steps identified by the meeting participants focused on an iterative phase of conversations with the community and regulatory and scientific bodies in order to develop a framework that is acceptable to all. We are currently planning a series of workshops with a range of key stakeholders to ensure optimal implementation in the country.

## Data availability

### Underlying data

Data provided in the manuscript may be used without request but with reference to the full article including the data. Other data will be made available with the approval of the OUCRU Data Access Committee (applications to ekestelyn@oucru.org), only where anonymization can be adequately achieved to protect the privacy and confidentiality of the participants and any mentioned individuals and institutions, and where the proposed use is seen as relevant to the nature of the data. Conditions for data sharing are outlined in the OUCRU data sharing policy available at http://www.oucru.org/data-sharing/.
