# Supplementary material for: Expert voices and equal partnerships: establishing Controlled Human Infection Models (CHIMs) in Vietnam
Source: Wellcome Open Res. 2019 Sep 25;4:143. [Version 1] doi: 10.12688/wellcomeopenres.15337.1 (PMC6820821; doi:10.12688/wellcomeopenres.15337.1)
Supplement: Peer review history

# Peer review: Reviewer response for version 1

**DOI:** 10.21956/wellcomeopenres.16745.r36574

**Published:** 2019-10-28

**Authors:** Seema K. Shah

**Affiliations:** 1 Lurie Children's Hospital, Department of Pediatrics, Feinberg School of Medicine, Northwestern University, Chicago, IL, USA

Thank you for the opportunity to review “Expert voices and equal partnerships: establishing Controlled Human Infection Models (CHIMs) in Vietnam". It is an interesting and well-written paper on an important topic. This article makes a valuable contribution to an understudied area. The concerns about community risk, benefit-sharing, and long-term risk from Dengue CHIMS in Vietnam are really interesting, as was the information that many clinicians felt there was exceptional local experience treating dengue that would likely reduce the risks and that the benefits could be significant. I have several suggestions that are not all essential to address, but that I hope can help clarify and strengthen the paper further.

*Introduction*:

 The language in the introduction could be a bit more careful. Because many associate the word “exploited” with the unethical treatment of research participants, it would be better to use different phrasing for the following sentence “Such studies have a long history and are increasingly being exploited to….” Additionally, the value of CHIMs go far beyond their use for diseases with *no* animal models, so I think the motivation for conducting CHIMs could be stated more strongly. Existing animal models for many diseases may have important limitations, and are rarely on their own sufficient for licensure. CHIMs can also accelerate the testing of new interventions, or be used when there is not enough ongoing transmission of a disease to study interventions in the field.

*Methods*:

 It is a bit unclear initially whether the primary data supporting the analysis in the paper is from the Global Forum meeting or the workshop in Vietnam. I think the Global Forum meeting was used to identify relevant issues that participants in the workshop in Vietnam were discussed, but a diagram demonstrating the different sources of data may be useful. To the extent Figure 2 is meant to do this, there is a lot of information in Figure 2 and the font is very small, making it harder to reconstruct how the authors drew out the themes they found. Also, finding a way to clearly distinguish whether any of the data in the discussion came from the GFBR meeting, or whether it is all from the workshop in Vietnam (which I think is the case) would be helpful. With regard to Figure 2, the codebook could simply be provided as an appendix that is available online for those who are interested.

*Results*:

 It would be helpful to be clearer about specific types of ethical issues that arise in CHIMs, and what the authors think of as an ethical issue. For instance, Figure 2 uses the terms “ethical considerations” or “ethics issues” in the lists of themes, even though other themes on the same list involve ethical issues (e.g., risk mitigation, risk-benefit assessment, community engagement). It may be that these terms are meant to refer to “special ethical issues”, but many authors have argued that CHIMs do not raise wholly unique ethical issues (see, e.g., Bambery *et al*. 2015 ^1^). Additionally, Figure 3 lays out subcategories for Scientific, Structural, and Cultural components but does not have subcategories for the Ethical component. Perhaps “Participant risk/benefit”, “Community risk/benefit” and “Participant selection” could be three sub-categories that are included under “Ethical”.

 Alternatively, the framework could be worked out in a different paper, and this paper could simply lay out questions and considerations that arose for doing CHIMs in Vietnam.

 I agree with reviewer 1 that more information about the views on a Shigella CHIM would be helpful. The emphasis in the current draft appears to be on Dengue CHIMs, and a separate section or Box on Shigella CHIMs would be helpful.

*Summary*:

 Some of the issues mentioned in the summary weren’t really presented in the results. It would be great to have more discussion of fair recruitment, informed consent, and compensation if there is more information to share about perceptions of these issues. Finally, instead of describing the potential framework to be developed as follows: “a framework that in principle allows a dengue CHIM to proceed”, it would seem not to presuppose a particular conclusion if the goal was described as follows: “a framework that can address the complex issues raised by a dengue CHIM in an endemic setting”.

I confirm that I have read this submission and believe that I have an appropriate level of expertise to confirm that it is of an acceptable scientific standard.

## References

[1] : Ethical Criteria for Human Challenge Studies in Infectious Diseases: Table 1. *Public Health Ethics*.2016;9(1) : 10.1093/phe/phv026 92-103 10.1093/phe/phv026 PMC592690429731811

# Peer review: Reviewer response for version 1

**DOI:** 10.21956/wellcomeopenres.16745.r36576

**Published:** 2019-10-28

**Authors:** Meta Roestenberg

**Affiliations:** 1 Department of Parasitology, Leiden University Medical Center (LUMC), Leiden, The Netherlands

This report outlines the discussion around Controlled Human Infection Models to be conducted in Vietnam. It provides a comprehensive overview of discussions which took place during a workshop around the topic in Vietnam. Although specific to the region and to dengue and shigella CHIMs in particular, the proposed initial framework can be used more broadly. The paper is very well written and clearly outlines the different ethical concepts.

 I have two general suggestions to further improve the paper for non-familiar readers:

- There are clear differences in risks between dengue and shigella CHIMs. To make the distinction clearer I suggest to provide a small overview of the risks specific to dengue as well as shigella CHIMs in the introduction section, preferably in a quantitative manner. In the summary, the differences or similarities between Dengue and Shigella CHIMs can be highlighted in view of the proposed framework
- In the introduction the potential scientific gain from dengue CHIMs in our increased understanding of the mechanisms underlying ADE should be stressed (independent of the use of the model to downselect vaccines), since in my view the scientific advance of dengue CHIMs could be transformative to the field of dengue research

Several minor comments:

- Under "individual participant risk" the last sentence reads "Some meeting participants also argued that ethical committees might not approve these types of studies under the criterion that personal risks for study subjects should not outweigh potential advantages to the wider community". An assessment of the balance between risks for the individual and benefits to the community is central to any review by an ethical committee, and certainly not unique to CHIMs. It thus is unclear to me what exactly the concerns of participants were.
- Under "immediate versus long-term risks" the last sentence "The possibility of... but there was concern that the risk window would continue indefinitely for volunteers in dengue-endemic areas". Did participants feel that this was unacceptable by definition, regardless of the height of the risk?
- Under "reimbursement and risk compensation", the reimbursement amounts are not discussed. Was this subject of debate at the workshop?

All in all, this paper is an important contribution to the ethical and regulatory framework of CHIMs in LMIC.

I confirm that I have read this submission and believe that I have an appropriate level of expertise to confirm that it is of an acceptable scientific standard.

# Peer review: Reviewer response for version 1

**DOI:** 10.21956/wellcomeopenres.16745.r36573

**Published:** 2019-10-02

**Authors:** Anna P. Durbin

**Affiliations:** 1 Bloomberg School of Public Health, Johns Hopkins University, Baltimore, MD, USA

I found this article to be very interesting and extremely important to the implementation of controlled human infection models in LMIC. The approach to laying a foundation for CHIM studies in Vietnam was well thought out and executed. The authors described their approach to introducing the concept of CHIMs to Vietnam as a multi-step process involving many different groups and processes. Their approach is one that can be utilized in other parts of the world that may be considering using CHIM. Although much of the paper is descriptive, it does present a framework for introducing the CHIM concept in LMIC where regulatory authorities may have no or very limited experience with this concept. The authors approach emphasizes the need to engage a diverse group of individuals from scientific, regulatory, government, community, and ethical backgrounds to educate and address concerns around CHIM studies. I thought the paper described these methods very well.

 I think it would be interesting if the authors discussed if the two CHIMs they brought forth had different acceptances? In Box 2, I wondered if the questions for the two different models were different because they got feedback from the first day and used that feedback to generate questions for the second day. I would revise Box 2 to put the Shigella questions first as they were asked on the first day and the dengue questions second as they were asked on the next day.

 The paper is extremely well written and will provide a strong blueprint for the introduction of CHIMs in other LMIC.

I confirm that I have read this submission and believe that I have an appropriate level of expertise to confirm that it is of an acceptable scientific standard.
